# Cohort-Specific Peptide Reagents Broaden Depth and Breadth Estimates of the CD8 T Cell Response to HIV-1 Gag Potential T Cell Epitopes

**DOI:** 10.3390/vaccines11020472

**Published:** 2023-02-17

**Authors:** Clive M. Michelo, Andrew Fiore-Gartland, Jama A. Dalel, Peter Hayes, Jianming Tang, Edward McGowan, William Kilembe, Natalia Fernandez, Jill Gilmour, Eric Hunter

**Affiliations:** 1 Center for Family Health Research Zambia, PostNet 412, P/Bag E891, B22/737 Bwembelelo, Emmasdale, Lusaka 10101, Zambia; 2 Vaccine and Infectious Disease Division, Fred Hutchinson Cancer Research Center, Seattle, WA 98109, USA; 3 IAVI Human Immunology Laboratory, Imperial College, London SW10 9NH, UK; 4 Department of Medicine, University of Alabama at Birmingham, Birmingham, AL 35294, USA; 5 Emory Vaccine Center, Emory University, 954 Gatewood Road NE, Atlanta, GA 30329, USA; 6 Emory National Primate Research Center, Emory University, 954 Gatewood Road NE, Atlanta, GA 30329, USA

**Keywords:** CD8 T-cell, epitope-mapping, HIV-1, HIV-1 vaccine

## Abstract

An effective HIV vaccine will need to stimulate immune responses against the sequence diversity presented in circulating virus strains. In this study, we evaluate breadth and depth estimates of potential T-cell epitopes (PTEs) in transmitted founder virus sequence-derived cohort-specific peptide reagents against reagents representative of consensus and global sequences. CD8 T-cells from twenty-six HIV-1+ PBMC donor samples, obtained at 1-year post estimated date of infection, were evaluated. ELISpot assays compared responses to 15mer consensus (*n* = 121), multivalent-global (*n* = 320), and 10mer multivalent cohort-specific (*n* = 300) PTE peptides, all mapping to the Gag antigen. Responses to 38 consensus, 71 global, and 62 cohort-specific PTEs were confirmed, with sixty percent of common global and cohort-specific PTEs corresponding to consensus sequences. Both global and cohort-specific peptides exhibited broader epitope coverage compared to commonly used consensus reagents, with mean breadth estimates of 3.2 (global), 3.4 (cohort) and 2.2 (consensus) epitopes. Global or cohort peptides each identified unique epitope responses that would not be detected if these peptide pools were used alone. A peptide set designed around specific virologic and immunogenetic characteristics of a target cohort can expand the detection of CD8 T-cell responses to epitopes in circulating viruses, providing a novel way to better define the host response to HIV-1 with implications for vaccine development.

## 1. Introduction

While much of the focus on a preventative vaccine has been on stimulating a broadly neutralizing antibody response, it is likely that the most effective vaccine will involve collaboration between both the humoral and cellular arms of the immune response. Indeed, recent studies in the SIV model showed that combining a neutralizing antibody response with a strong cellular response to Gag antigen resulted in superior and more durable protection [1,2]. Cytotoxic T-lymphocyte (CTL) mediated immunity has been shown to be critical for the elimination of HIV-1 infected cells and for the containment of acute infection. The development of effective T cell responses is associated with a reduced rate of disease progression [3,4,5,6]. Specifically, the development and maintenance of a polyfunctional HIV-1 specific CD8 T cell response is critical to viral control [7,8,9,10] and is currently the focus of several vaccine candidates designed to stimulate HIV-1 specific CD8 T cells. The identification of CD8 T cell epitopes within HIV-1 is important for understanding the underlying mechanisms of T cell immunity and thereby informing on prophylactic or therapeutic vaccine design. In vivo, viral peptides are presented to CTLs bound on major histocompatibility class I (MHC-I) molecules, thereby dictating with their binding specificity the potential repertoire of CTL responses. Thus, it is important to understand the MHC-I restriction of CTL responses and its role in host immunity and viral diversification [11,12,13,14].

HIV-1 genetic diversity poses a major barrier to both the design and evaluation of HIV-1 vaccines. An effective vaccine will need to elicit broadly reactive immune responses [15,16]. Although the use of vaccine modalities based on conserved elements [17], or central sequences (ancestral or population consensus sequences [18,19]), enabled improvement in the breadth (number of discrete epitopes) of HIV-specific T cell responses elicited, they still show limited ability to elicit responses that recognize the sequence diversity of circulating HIV [20]. Alternatively, multivalent sequences are currently being employed as vaccine immunogens which, in addition to breadth, also increase the depth of the response (number of recognizable epitope variants), and therefore, improve the ability of vaccine recipients to broadly recognize potential exposures [21,22,23,24]. However, the evaluation of such responses with vaccine-matched peptides may fail to fully characterize the depth of the T cell response to circulating variants [25]. Therefore, for the rational development of a T cell-based HIV-1 vaccine, a better approach to screening and identifying potential T cell epitopes (PTEs) is required.

To better evaluate the breadth and depth of T cell responses in individuals living with HIV-1 or vaccinated individuals, a set of 15mer peptides were designed to provide optimal coverage of global circulating viruses, as represented by sequences deposited in the LANL database in 2006 [26]. The resulting global peptide set included variant peptides that were representative of circulating viruses and specifically excluded peptides that were unlikely to represent epitopes based on analyses of 1900 HIV-1 epitopes that were known at the time; these peptides overcame some of the limitations of peptides based on the consensus sequences for specific subtypes [26,27,28]. However, viral sequences from contemporary individuals living with HIV-1 in a specific population may still be poorly represented by these global PTEs. 

While autologous peptides representing the Gag sequence of each participant in a study would optimally probe the extent of CD8 T cell responses, this is prohibitively expensive. We, therefore, developed an alternative to the global PTEs for epitope mapping of a specific cohort by applying a similar peptide coverage optimization algorithm but substituting the LANL database sequences with inferred transmitted founder virus (TFVs) sequences defined from Zambian individuals with acute clade C HIV-1 infection. Furthermore, the algorithm uses the HLA-alleles of each individual to discard peptides that are not predicted to bind class I MHC, using state-of-the-art computational epitope prediction (NetMHCpan 2.8) [29]. By selecting PTEs (cohort specific) from the participants’ TFV sequence variants and excluding non-MHC-binding peptides (IC_50_ > 10 mM) we hypothesized that we could more effectively identify epitopes and their variants compared to the existing global PTE approach. To test this hypothesis, we evaluated T cell responses by ELISpot to a library of 794 cohort, global and consensus PTEs, using a hierarchical matrix pool strategy that significantly reduced the number of tests and cells required without compromising the specificity of the ELISpot procedure. Our data showed that cohort-specific PTEs designed from viral isolates from local study participants living with HIV yielded increased estimates of epitope breadth for the immune response relative to consensus peptides and enabled a unique estimation of depth that can further expand estimates of approaches using global peptides.

## 2. Materials and Methods

### 2.1. Study Participants

All samples were collected under International AIDS Vaccines Initiative (IAVI) sponsored study protocols conducted at Zambian research centers. PBMC samples were isolated from blood samples collected with written informed donor consent. A sampling of 30 HIV-1-infected participants was made approximately 365 days post their Estimated Date of Infection (EDI) (Table 1).

**Figure 1:**
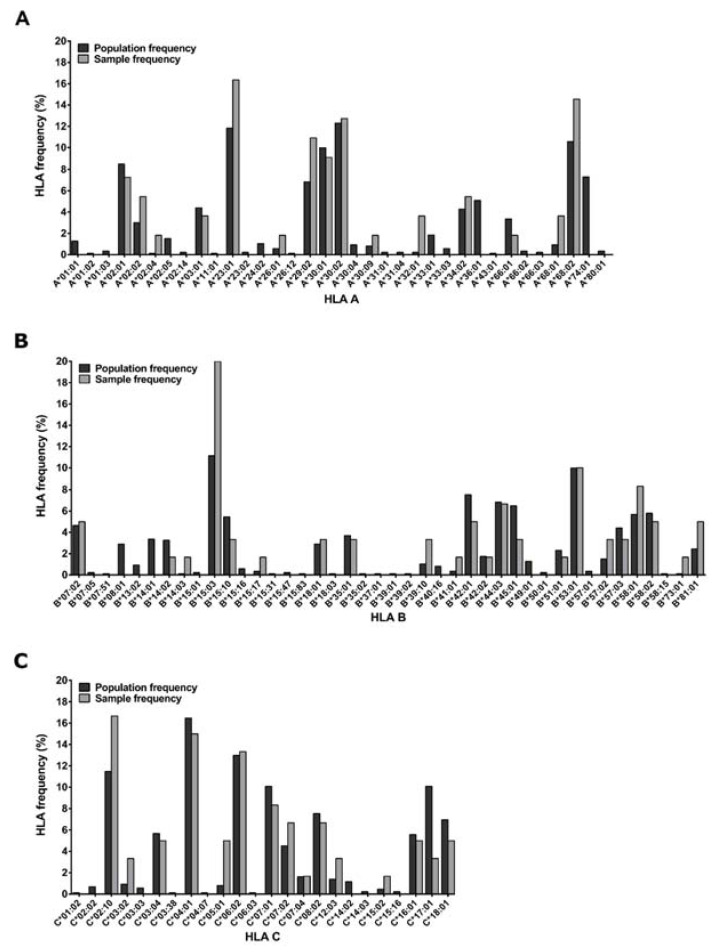
HLA of the 30 sample donors. Comparison of HLA frequencies of the subset of PBMC donors used in the study and the larger Zambian source participant population (*n* = 233). HLA genotyping was conducted on genomic DNA extracted from whole blood or buffy coats using PCR for two-digit specificity.

Participants living with HIV were part of the IAVI-sponsored acute infection study cohort called Protocol C, where over 3000 time points were sampled for plasma and PBMCs from 286 Zambian participants collected at the Zambia-Emory HIV Research Project (ZEHRP) laboratories in Lusaka and Ndola. Protocol C was a prospective, observational, multi-center study conducted between February 2006 and December 2011 to evaluate laboratory, clinical, immunologic and viral markers of disease progression in participants who had recently acquired HIV [30,31,32]. HLA genotyping of 233/286 Protocol C Zambian participants was performed using genomic DNA extracted from whole blood or buffy coats (Qama blood kit; Qiagen, Hilden, Germany) using PCR with commercial sequence-specific primers for two-digit specificity as described previously [33], (Figure 1). The study was reviewed and approved by the Emory University Institutional Review and University of Zambia Research Ethics Committee (Emory IRB00000737) and all participants consented to participate in the study following sessions to fully inform them of its requirements and goals. 

### 2.2. PBMC Processing

Fresh PBMC were separated from whole blood by Ficoll-Hypaque (Histopaque^®^-1077 Hybri-Max™) density gradient centrifugation and frozen in liquid nitrogen using fetal bovine serum (FBS) supplemented with 10% dimethyl sulphoxide (DMSO) at a concentration of 10 × 10^6^ cells/vial. PBMC were thawed with culture medium; RPMI 1640 medium supplemented with penicillin (100 U/mL), streptomycin (100 μg/ mL), L-glutamine (2 mM), sodium pyruvate (1 mM), HEPES buffer solution (10 mM) and 10% or 20% FBS (R10 or R20 media, respectively). All reagents were supplied by Sigma, St. Louis, MO, USA.

### 2.3. CD8 T Cell Expansion

To extend clinical sample availability, CD8 T cells were expanded as previously described [34,35,36,37]. Briefly, thawed PBMC were cultured initially at 1.0–1.5 × 10^6^ cells/mL in R10 with IL-2 at 50 U/mL (R10/50) and CD3/CD4 bi-specific antibody at 0.5µg/mL at 37 °C and 5% CO_2_. The volume of culture medium was doubled at days 3 and 5 of cell culture with R10/50. On day 7 of culture, cells were counted and approximately 9 × 10^6^ cells per set of peptide pools to be tested were removed from culture for the first ELISpot with peptide pools. The cells were washed of IL-2 and rested for 22–26 h in R10 without IL-2 before plating for the first ELISpot. The remaining cells were cultured in R10/50 at 37 °C and 5% CO_2_ until day 10 when all cells were harvested, washed and rested as before, before plating for the second ELISpot with individual peptides.

### 2.4. HIV-1 Gag Peptide Sets and Pools

Three sets of HIV-1 Gag protein peptides were used in this study. The first set consisted of 121 Consensus clade C Gag 15mer peptides with an 11 aa overlap between subsequent peptides. These covered the whole length of Gag (NIH AIDS reagents program cat. 8118: HIV-1 Consensus C Gag Peptide Set and cat. 12756: HIV-1 Consensus C Gag Peptide Pool). A second set of 320 15mer peptides, termed “global” PTEs, were generated from sequences in the LANL database in 2006 [26], (NIH AIDS reagents program cat. 11554: HIV-1 PTE Gag Peptide Set and cat. 12437: HIV-1 PTE Gag Peptide pool). A final peptide set, termed “cohort” PTEs, consisted of 300 10mer peptides designed from the TFV Gag sequences of 127 Zambian protocol C participants. Zambian HIV-1 seroconverters were identified with a median (interquartile range) estimated time since infection (ETI) of 46 (42 to 60.5) days, at which time plasma samples were obtained and the *gag*-gene sequence of the transmitted virus was determined as described previously [38]. The cohort PTEs were extended to include 53 peptides from the “A list” of best-defined CTL epitopes (https://www.hiv.lanl.gov/content/immunology/tables/optimal_ctl_summary.html, assessed on 31 December 2017) described by Llano et al. 2013 [39], that were not already identified by the algorithm.

Cohort-specific PTEs were designed using an adaptation of the PTE algorithm (Li et al., 2006) that was used to design the global PTEs, which are publicly available from the NIH. Both algorithms work by selecting peptides to provide optimal coverage of an amino-acid sequence alignment. In the original approach, from the alignment two sets of peptides are generated: (A) all 9mers representing potential CD8+ T cell epitopes, (B) all unique 15mers forming a pool of peptides to pick from. Peptide selection begins by selecting a 15mer from (B) that provides maximal coverage of 9mers in (A), based on their frequency in the alignment. Naturally, 15mers covering conserved 9mers are selected first. Selection proceeds by removing the “covered” 9mers from (A) and picking the next best 15mer from B that provides maximal coverage. The selection continues until the desired number of PTEs is attained or until the marginal gain in coverage falls below a pre-specified threshold (i.e., stop selection when the next peptide would provide less than 10% additional coverage of epitopes in the alignment).

The Cohort-specific PTE algorithm was adapted from this original algorithm by further restricting (A) to 9mer peptides that were predicted to bind an HLA allele with IC50 < 10,000 nM using NetMHCpan (v2.8). We intentionally used an inclusive IC50 threshold because we noted that many known HIV-1 epitopes in the LANL database have predicted IC50 > 500 nM, a more typical threshold. Using a higher threshold ensures that most epitopes will be covered by the cohort-specific peptides, while still allowing for substantial efficiency gains: ~70% of 9mers had an IC50 > 10,000 nM and could, therefore, be excluded from (A). The cohort-specific PTE peptides were created from an alignment of 127 TFV clade C sequences isolated from individuals in Zambia (Protocol C); the matching HLA-A and HLA-B alleles of each participant were used to generate the starting pool of potential epitopes (A). We used 10mer peptides, extracted from the collection of the 127 TFV clade C viral sequences and selected a sub-set of these that covered the target 9mer PTEs.

The panels of consensus, cohort and global peptides were organized into a 15-pool 3D matrix for the consensus peptide set while the cohort and global peptide sets were organized into 18-pool 3D matrices. The organization was such that, within a given peptide set matrix, each peptide was represented in three different peptide pools and the pools were positioned one at each matrix axis. This allowed the identification of the respective peptide by responses in the three corresponding pools. The consensus pool composition ranged between 21 and 25 peptides per pool (median of 24 peptides per pool). Peptide pool composition for the cohort and global peptide sets ranged between 50 and 74 peptides per pool (median of 58 peptides per pool) and 43 and 63 peptides per pool (median of 52 peptides per pool), respectively. The final assay concentration of each peptide within a peptide pool as well as in single deconvoluted single peptide runs was 2.0 µg/mL in 150 µL of assay volume. 

### 2.5. ELISpot Assays and Mapping Strategy

IFN-γ ELISpot assays were performed on cryopreserved PBMCs using previously detailed methods using a peptide pool matrix-screening step with a subsequent deconvoluted individual peptides analysis step [34,35,40]. Polyclonally expanded CD8 T cells extended sample availability for the three peptide sets covering the HIV-1 Gag protein using the ELISpot assay. The ELISpot assay procedures have been previously described [41]. Briefly, 7-day polyclonally expanded and 24 h rested CD8 T cells were recovered, viably counted (Beckman Coulter Vicell XR) and plated in duplicates in 96-well anti-IFNγ pre-coated flat bottom PVDF culture plates [42], (Mabtech, Nacka, Sweden) at 2 × 10^5^ cell per well with stimuli. A no-cell well was used to access the medium background. Non-specific IFNγ secretion by CD8 T cells was assessed by culture with 0.45% dimethyl sulfoxide (DMSO) (Sigma) in R10 (mock). Positive control stimuli were phytohemagglutinin (PHA, 10µg/mL) (Sigma) or cytomegalovirus (CMV) pp65 protein peptide pool. Plates were incubated overnight at 37 °C, 5% CO_2_ and spots developed as described previously [41]. ELISpots using single peptides deconvoluted from the day-7 CD8 T cells matrix pool ELISpots were performed on day 10 expanded CD8 T cells as above. The day 10 CD8 T cell single peptide assay included a confirmatory re-testing of the day 7 CD8 T cell positive pools. The numbers of spot forming units (SFU) per well were counted using an automated ELISpot plate reader (AID ELISpot reader system; Autoimmune Diagnostika GmbH, Strassberg, Germany) and the number of specific T cells was calculated by subtracting the mock control values. SFU were expressed as mock corrected SFU per 10^6^ cells, i.e., the average mock SFU was subtracted from the observed pool or single peptide SFU values. Based on our previous work [41,42] on assay validation using PBMCs, a mock subtracted response of ≥38 SFU/10^6^ CD8 T cells was scored as a positive response to a pool or peptide. 

### 2.6. Statistical Analysis

Sequence alignment and analysis were performed using sequence analysis programs Jalview V.2 [43], and MEGA7 [44]. Statistical analysis and graphical presentation were conducted using GraphPad Prism v6,01 for Microsoft Windows and Python programming language (Python Software Foundation. Python Language Reference, version 3.6. Available online: http://www.python.org; accessed on 1 September 2018). Statistical results are given as means with standard deviation (SD) or 95% confidence intervals and significance (*p* values) based on two-tailed t-tests or medians with the interquartile range and significance (*p* values) as required by the test. Correlation comparisons were made using the Spearman rank correlation test and linear regression analysis was used to determine the slope. 

## 3. Results

### 3.1. Epitope Mapping of CD8 T Cell Responses

**Figure 2:**
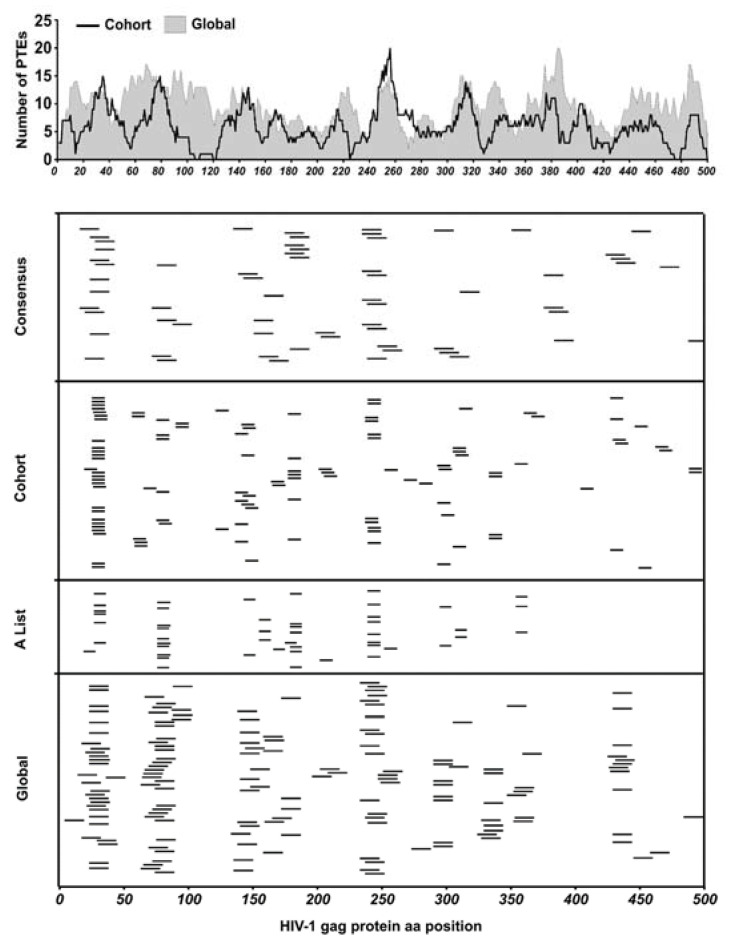
Peptide coverage and response epitope map. (**Top** panel) All peptides in global and cohort PTE sets were aligned to the HIV-1 HBX2 Gag protein sequence to estimate the number of peptide sequence variants covering the sequence positions of the Gag protein by the global and cohort peptide sets by counting the number of amino acids aligned at each position. (**Bottom** panels) Each peptide from the different peptide sets used in this study that elicited a positive CD8 T cell response was mapped to the HIV-1 Gag protein sequence to determine their relative locations along the Gag protein.

We evaluated the performance of three sets of HIV-1 Gag-derived peptides in epitope mapping experiments: (1) clade C consensus peptides (2) Global PTEs derived from mixed clade Gag sequences and (3) HIV-LANL “A list” HIV epitope supplemented Cohort PTEs derived from TFV Gag sequences. The peptide design approach to the global and cohort multivalent peptides was similar with the major difference being the input sequences. The three peptide sets together were comprised of 794 peptides which we used to identify epitopes targeted by polyclonally expanded CD8 T cells. With these sets of peptides, we tested our hypothesis that using peptides derived from contemporary, cohort-specific TFV sequences compared to globally circulating sequences would enable more effective identification of T cell epitopes and further allow the better estimation of response depth. By comparatively aligning all the global and cohort PTE peptides to the HBX2 HIV-1 Gag protein sequence we estimated the number of sequence variants covering the HBX2 sequence positions by counting the number of amino acids aligned at each position, with global and cohort showing an average of nine and seven amino acids per position, respectively (Figure 2 upper panel).

Epitopes were mapped in two subsequent ELISpot assays; the first ELISpot screened large peptide pools organized into 3D matrices, while the second tested individual peptides deconvoluted from the pool matrices as we have described previously [34,35]. ELISpot on deconvoluted peptides identified 38/121 consensus, 71/320 global and 62/300 cohort-specific reactive peptides from 26/30 participants living with HIV in the cohort. Each ELISpot-positive peptide was mapped to the HBX2 HIV-1 Gag protein sequence to determine their relative locations (Figure 2 lower panels). As is typical of individuals living with HIV, there were several epitope hotspots targeted by two or more participants, along with several less immuno-prevalent epitopes targeted by a single participant [45].

**Figure 3:**
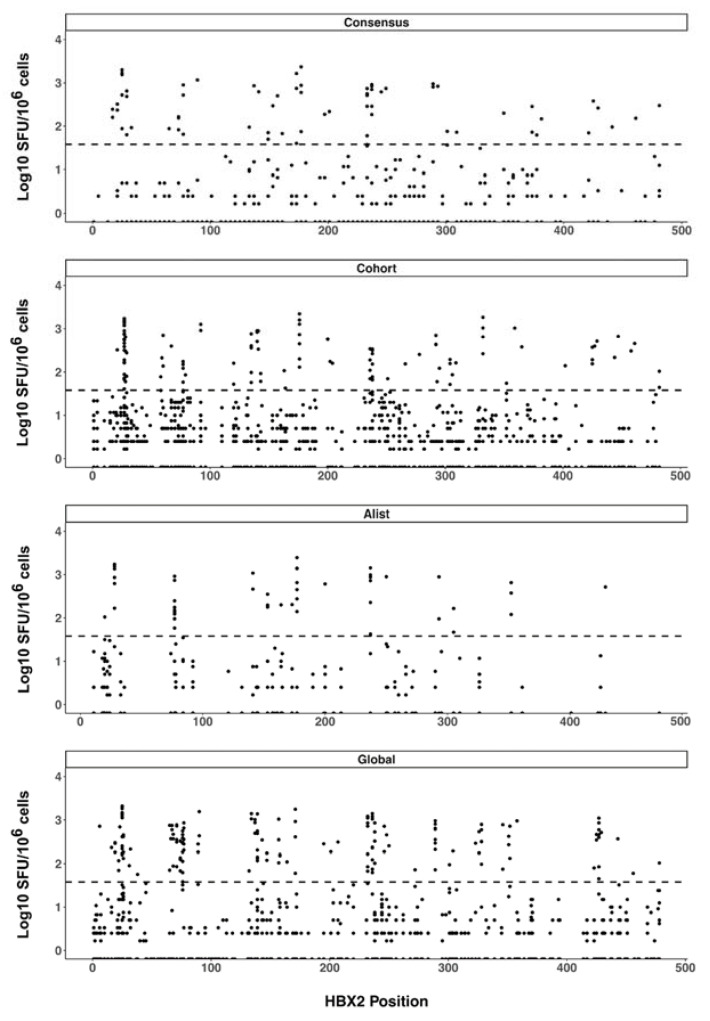
CD8 T cell response magnitude. ELISpot response magnitudes were plotted according to the location of the start amino acid of the response peptide along the HIV-1 Gag sequence. Each spot represents the response magnitude CD8 T cells mapped to the relative location of the peptide on HIV-1 Gag. The dashed line indicates the positive response threshold (38 SFU/10^6^ or 1.58 Log10 SFU/10^6^).

We also plotted all the peptide response magnitudes according to their HXB2 amino-acid start position (Figure 3). The resulting response magnitude profiles did not indicate any apparent particular Gag region targeting bias in the CD8 T cell responses beyond the observed epitope hotspots. Considering only peptide responses with magnitudes above the pre-specified threshold for the positive response of 38 SFU/10^6^ cells (1.58 Log10 SFU/10^6^ cells), there was no significant difference in the magnitudes of T cell responses to any of the pairwise comparisons of the four different peptide sets (SFU/10^6^ CD8 cells mean ± SD [Log10 SFU/10^6^ cells], consensus 467.3 ± 495.86 [2.67 ± 2.70], A-list 573.8 ± 541.73 [2.76 ± 2.73], cohort 425.7 ± 451.38 [2.63 ± 2.65] and global 485.6 ± 453.17 [2.69 ± 2.66]).

### 3.2. Epitope Identification and Analysis of Breadth

T cell epitope breadth can be defined as the number of distinct regions of a protein or pathogen that are targeted by an individual and is an immunogenicity endpoint that has been used widely in HIV-1 vaccine clinical trials as well as natural infection studies to understand the mediators of viral control. Epitope breadth can be estimated from epitope mapping experiments; however, counting the number of peptides that elicit a significant ELISpot response is typically an overestimate of breadth when overlapping peptides are used in the experiment. Instead, we defined the minimal epitope breadth of a single participant as the minimum number of distinct peptide regions, each between 8–15 contiguous amino acids, that can explain all of that participant’s positive ELISpot responses.

To estimate breadth, we employed an exhaustive search algorithm to identify combinations of a participant’s peptide responses that could be explained by a single epitope by applying an overlap criterion where if two or more peptides share a region of ≥8 positions then they can be explained by a single epitope (Supplementary Figure S1). This criterion was applied iteratively to all possible combinations of each CD8 T cell sample’s peptide responses, to estimate the minimal epitope breadth yielding the lowest number of epitopes that could explain all the responses. It is likely that this procedure provides an underestimate of the actual number of distinct regions targeted by CD8 T cells. For example, if a response to a single 15mer peptide contains two 9mer regions at the N and C termini targeted by different T cells, the algorithm will count the 15mer as a single epitope. Such a response could be parsed as two distinct epitopes if additional overlapping 15mers each spanned only one of the epitopes at the N- and C- termini. The algorithm also attributed each peptide response to one or more epitopes, based on an overlap of at least eight amino acids. Thus, for each epitope, the putative optimal epitope was determined to be the region overlapping all of the peptide responses attributed to the epitope. For the majority of epitopes, we were able to use peptide overlap to determine an epitope region of between 8–11 amino acids. For each epitope, the optimal length was determined based on the maximum length of consecutive amino acids overlapping all of the assigned peptides (Supplementary Figure S2) excluding “floater” peptides, whose response could not be attributed definitely to a single epitope. When multiple proximal epitopes are required to explain a set of responses, there are occasionally floater peptide responses that could be attributed to one or both of the epitopes; in other words, it was unclear which part of the peptide contained the optimal epitope.

**Figure 4:**
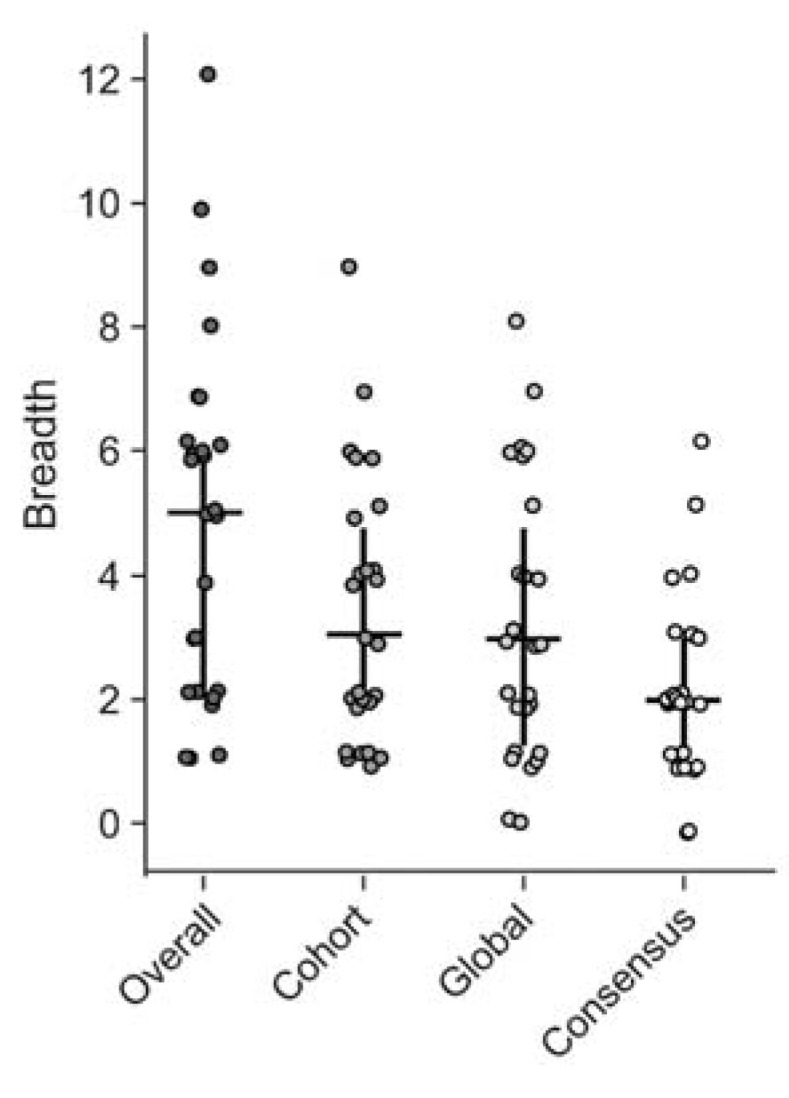
CD8 T cell Response breadth estimation. Minimal epitope breadth of T cell responses as determined using either the combined (overall), cohort PTE, global PTE or consensus peptide sets. Responses to consecutive peptides overlapping by 8aa were counted as a single epitope. Vertical lines indicate the interquartile range (IQR) with a horizontal line indicating the median. Circles indicate the minimal epitope breadth of each subject.

We first estimated the minimal epitope breadth for each participant using all the 15mer and 10mer peptides that were identified as significant responses by the epitope mapping procedure (overall) (Figure 4). Then we re-analyzed the data as if only global PTE, cohort PTE or consensus peptides had been used for epitope mapping, to compare their respective estimates of epitope breadth (Figure 4). We found, using the peptides from all three peptide sets, that participants targeted a range of 1 to 12 distinct epitopes in Gag; mean overall breadth was 4.9 (95% CI [3.8, 6]). Using either the global (3.2 [2.4, 4.1]) or cohort PTEs (3.4 [2.6, 4.2]) alone resulted in lower estimates of breadth (Wilcoxon signed-rank *p* < 0.001), suggesting that there were epitopes detected exclusively by each set and demonstrating that generally, as would be predicted, epitope mapping with more peptides will tend to increase the estimate of minimal epitope breadth. There was no significant difference between the breadth estimated from global and cohort peptides (*p* = 0.42). Using only consensus peptides yielded breadth estimates for each participant that were significantly lower than the other peptide sets or the combined peptides (2.2 [1.7, 2.7], all *p* < 0.001). 

### 3.3. Assessment of Epitopes Detected by Global or Cohort Peptides or Both

**Figure 5:**
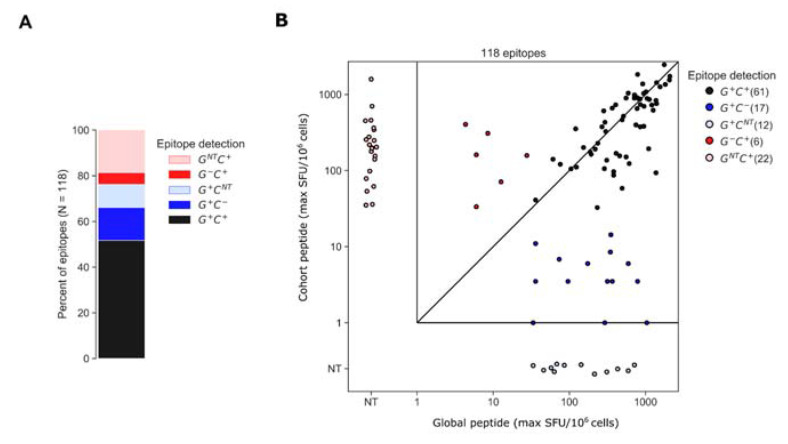
Epitopes identified by global and cohort peptide responses. (**A**) Epitopes were categorized based on whether they were detected by both global and cohort peptides (G^+^ C^+^), global-only (G^+^ C^−^ or G^+^ C^NT^) or cohort-only peptides (G^−^ C^+^ or G^NT^ C^+^). If no peptides overlapping the epitope were tested from a given set, due to negative pool-level responses the epitope is indicated as not-tested (NT) for that set (**B**) Plot indicates the maximum magnitude of the peptide responses among all peptides overlapping the epitope from each peptide set. Maximum response magnitudes among G^+^ C^+^ epitopes were positively correlated (R = 0.71, *p* < 0.001).

To better understand why certain epitopes were detected exclusively using global or cohort peptides, we categorized each participant’s epitopes based on whether each was contained within global or cohort peptides; for this analysis, we ran the epitope identification algorithm using global and cohort peptide responses combined. The algorithm identified 118 epitopes among 26 of the 30 participants. Of the 118 epitopes, 61 (52%) were detected by both global and cohort peptides (G^+^ C^+^) and of the 57 remaining epitopes, 29 (28) were detected exclusively by global (cohort) peptides (Figure 5). The “global-only” epitopes were further categorized as either sub-threshold cohort responses (G^+^ C^−^ [17 epitopes]) or cohort peptides that were not tested (NT), due to a negative pool-level result (G^+^ C^NT^ [12 epitopes]). Likewise, the “cohort-only” epitopes were a combination of epitopes that were detected by cohort peptides but were not detected by global peptides because overlapping peptides either elicited sub-threshold responses (G^−^ C^+^ [6 epitopes]) or were not tested due to a negative response at the pool level (G^NT^ C^+^ [22 epitopes]) (Figure 5). For each of the G^+^ C^+^ epitopes, we plotted the maximum ELISpot magnitude (SFU/10^6^ cells) among the positive peptides from either the global 15mers or cohort 10mers; the magnitudes were well correlated (rank correlation, R = 0.71, *p* < 0.001) though global peptides tended to elicit higher maximum magnitudes than cohort-specific peptides on average; however, the difference was statistically insignificant (SFU/10^6^ CD8 cells mean ± SD [Log10 SFU/10^6^ cells], cohort 671.77 ± 545.57 [2.82 ± 2.74] and global 765.36 ± 544.25 [2.88 ± 2.73], *p* = 0.40).

### 3.4. Analysis of Epitope Response Depth

**Figure 6:**
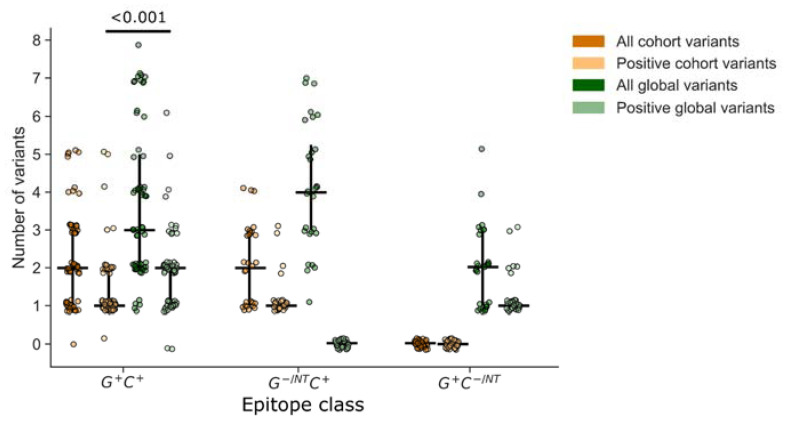
T cell response depth. Response depth was estimated by counting the number of peptides with unique sequence variations within the putative epitope region. For each peptide set, global vs. cohort, we counted the total number of unique peptide variants and the number that were contained within at least one peptide that tested positive. Each symbol represents one epitope, recognized by one participant (n = 118 epitopes). Vertical lines indicate the IQR with horizontal lines indicating the median. Epitopes are categorized based on their identification by at least one positive peptide from each set (G^+^C^+^), or from one set and not the other (G^−/NT^C^+^ or G^+^C^−/NT^).

The depth of a T cell response can be an important metric indicating the utility of a T cell response in cross-recognizing multiple peptide variants. We defined depth as the number of peptide variants recognized by an individual within the epitope-defined region and compared this to the total number of peptide variants overlapping the epitope region in each peptide set. First, we compared depth among epitopes that were detected using both global and cohort peptides (G^+^C^+^; Figure 6). By definition, the total number of peptide variants included for a given epitope was greater than or equal to the number that tested positive. Overall, both the global and cohort peptide sets provided the ability to detect responses to several variants of each epitope (depth), with global detecting a higher median number of variants than cohort peptides (*p* < 0.001), which could be partly attributed to the greater number of variants per epitope that were included in the global peptide set. However, the comparison of relative proportions indicated that fewer included variants were recognized in the global (59%) compared to the cohort (75%) peptide sets, suggesting that the global peptides were less efficient at detecting variant responses. Consistent with this observation we noted that among the epitopes that were detected using cohort peptides only (G^−^ C^+^), there were more variants per epitope represented in the global (median 4) versus cohort (median 2) peptides, yet no recognition of any of the global peptide variants. In contrast, the global-only responses (G^+^ C^−^) could be explained by a lack of coverage in the cohort peptide set, not the lack of a specific variant.

### 3.5. Importance of Non-Consensus Peptides in Epitope Mapping 

**Figure 7:**
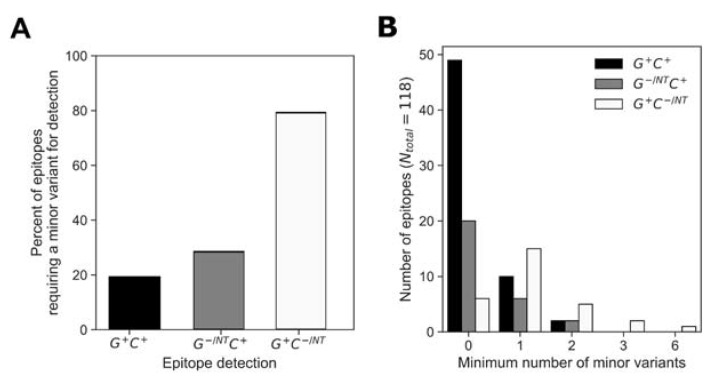
Consensus AA composition in the global and cohort peptides responses. We analyzed for instances when at least one minor variant was required for response detection (**A**) and categorized the determined epitopes in the G^+^ C^+^, G^+^ C^−^ and G^−^ C^+^ groups to minimum number of minor variants required for detection (**B**).

The low breadth in responses to consensus peptides indicated that the consensus peptides were not sufficient for an accurate estimation of response depth. At almost all positions, the consensus amino acid was represented in both the global and cohort peptide sets; however, the consensus variant peptide was not always recognized for a given epitope. We assessed for each epitope whether or not a non-consensus peptide was required for epitope identification and if so, the minimum number of amino acid substitutions relative to consensus that were required for recognition (Figure 7). Analysis of the epitopes that were detected using both the global and cohort peptides (G^+^ C^+^) showed that 81% of the determined epitopes did not require amino-acid substitutions relative to consensus (49/61 G^+^ C^+^ epitope AA were wholly consensus) (Figure 7A). Of the remainder of the G+ C+ epitopes (20%), 10/61 required at least one AA substitution for detection, while 2/61 required two AA substitutions relative to the consensus sequence (Figure 7B).

Similarly, of the epitopes that were detected by cohort and not global peptides (G^−/NT^ C^+^), about 70% were detected using a peptide sequence that matched the consensus (i.e., 20/28 G^−/NT^ C^+^ epitope AA were wholly consensus) while 6/28 G^−/NT^ C^+^ epitopes required at least one AA substitution and 2/28 G^−/NT^ C^+^ epitopes required two AA substitutions relative to the consensus sequence for detection. However, only 20% of epitopes that were detected only by global peptides (G^+^ C^−/NT^) did not require amino-acid substitutions in order to be detected, 6/29 G^+^ C^−/NT^ epitopes were wholly consensus, 15/29 (52%) G^+^ C^−/NT^ epitopes required at least one AA substitution and 5/29 G^+^ C^−/NT^, 2/29 G^+^ C^−/NT^ and 1/29 G^+^ C^−/NT^ epitopes required at least two, three or six AA substitutions, respectively). In summary, 75/118 (64%) of the determined epitopes were wholly AA consensus sequences while the remaining 43/118 epitopes required one or more AA substitutions relative to the consensus sequence to be detected. Further analysis of the magnitudes of responses showed that the epitopes requiring a non-consensus peptide did not have significantly different response magnitudes from those that were detected by a consensus peptide (Supplementary Figure S3).

### 3.6. Accounting for Missed Responses

Cohort and global peptides were able to detect a similar number of epitopes (i.e., similar breadth). However, there were many epitopes detected by one peptide set and not the other. In these cases, we investigated whether the lack of response to peptides from one set could be attributed to a hole in positional coverage or a missing variant required for recognition since the occurrence of as little as one amino acid substitution can abrogate T cell receptor recognition [46].

We began by assessing the global-only epitopes that were not detected by cohort peptides. Though many of these epitopes were detected by more than one global 15mer, making it possible to identify a putative epitope region of overlap within the 15mers, none of the regions were <11 amino acids, making it difficult to determine the exact epitope and potentially complicating the attribution of a missed response (Supplementary Figure S4). Therefore, we asked how many of the 9mers within positive global 15mers were covered positionally by cohort peptides and how often did the 9mers match a cohort peptide sequence exactly. We found that 17% of global peptides within G^+^ C^−/NT^ epitopes were fully covered by cohort peptides and that there was 77% coverage on average. However, only 35% were covered by sequence-matched cohort peptides. Together these data suggest that responses missed by cohort peptides could be explained both by missing amino-acid variants and holes in coverage.

On the other hand, we found that 89% of cohort-only epitopes were completely contained by at least one global peptide. Furthermore, for 79% of these epitopes, there was a global peptide that matched the sequence of at least one cohort peptide that was recognized, suggesting that some factor other than a missing variant or missing coverage may explain these missed responses.

### 3.7. Epitope Location within Global 15mer Peptides

**Figure 8:**
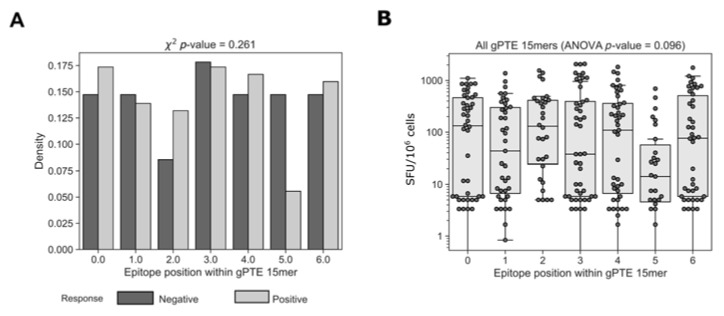
Accounting for responses missed by various peptide pools. Relative location of the determined epitopes was compared between positive and negative 15mer global peptides. Further analysis was also conducted on epitope location categorized response magnitudes. Peptides that were missed by one and not the other peptide sets were categorized according to the variance characterizing the AA overlap between the epitope and the negative or not tested (NT) peptide.

One possible explanation for epitopes that were not detected by global 15mers could be related to the position of the epitope within the peptide. The use of 15mer peptides for the detection of CD8+ T cell responses is a compromise between presenting a peptide close to the typical CD8+ MHC class I-restricted epitope (typically 8–11mer) and a peptide long enough for the presentation of more than one epitope per peptide. This, however, presents a potential disadvantage in the possibility that epitopes situated in the middle of longer peptides may not be detected because of suboptimal epitope presentation [47]. To determine whether the position of the epitope within a global 15mer impacted recognition we compared the position of the epitopes detected by cohort peptides within positive and negative overlapping global 15mers (Figure 8). We did not find any difference in the location of the epitopes within 15mers that elicited a positive or negative response (c^2^ *p* = 0.261). We then compared the magnitude of T cell responses organized by epitope position; there was no significant difference in the response to epitopes that started at different positions within the 15mer (ANOVA, *p* = 0.096). Together these results show that there was no general rule governing the role of epitope location on T cell recognition; however, it does not eliminate the possibility that for specific epitopes location within a 15mer is important. 

## 4. Discussion

Extensive genetic global HIV-1 diversity is one factor, among others, that has hindered the design and development of an efficacious HIV vaccine [48,49]. It is now well appreciated that to develop an effective and preventative HIV vaccine that would have a significant role in the control of the spread of HIV, the successful vaccine candidate will have to be able to elicit broad immunogenicity against the diversity represented in circulating strains [26]. Hence, epitope mapping is an important way to identify cellular responses associated with viremic control in natural infection and a way to evaluate vaccine immunogenicity. Ideally, epitope mapping would be conducted by testing all potential epitopes from an individual’s autologous quasi-species or from the pool of potential exposures, but the virtually unlimited number of potential variants prohibits this approach. One approach to address this problem has been to use consensus peptides, which are considerably more limited in number and are able to identify responses that recognize conserved regions but fail to capture the diversity of responses to non-conserved regions. An alternative approach has been to use a set of multivariant peptides derived from a global 2006 database of sequences, striking a balance between the constraints of an epitope mapping experiment and the potential variants that may be recognized by an individual. Here, we sought to develop an approach for selecting peptides that would be optimized for identifying epitopes within a specific cohort of individuals living with HIV or importantly, within a vaccinated cohort at risk of infection from a specific set of circulating HIVs. To evaluate this approach as well as map for early time point viral antigenic targets, we used TFV sequences from a cohort of individuals to generate a set of HIV-1 Gag PTE peptides (cohort PTEs) that were used to map early time point T cell epitopes specific to the virologic and immunologic characteristics of the population. We show that cohort PTEs enabled the detection of both consensus epitopes and epitopes that contained minority variant amino acids, resulting in increased estimates of epitope breadth relative to consensus peptides and enabling the estimation of response depth. In many cases, this increase in recognition of responses using non-consensus peptides could be directly tied to a non-consensus amino acid sequence variant.

The direct comparison of global 15mer PTEs and cohort 10mer PTEs showed no significant difference in the number or magnitude of the responses recognized. However, the comparison of different-length peptides is a limitation of the study. A majority (81%) of the responses elicited by both peptide sets were also elicited by consensus peptides, demonstrating the value of consensus peptides for epitope mapping of HIV-1 Gag antigen responses [50,51]. However, nearly 50% of responses were elicited by only the global or cohort peptide sets. We found that global peptides were typically able to elicit a response if they overlapped the identified epitope region; missed responses were more likely to be related to holes in positional coverage as opposed to missing sequence variants. In contrast, the responses that were elicited exclusively by cohort peptides were often positionally covered by a global 15mer peptide and missed responses were attributable to cohort-specific variants that were not represented or a lack of response despite having a matched overlap. Further, examination of the 15mers that failed to elicit a response despite an exact match with a cohort peptide-defined epitope did not identify a general pattern of epitope location underlying the failed recognition; this is contrary to previous results suggesting that epitopes situated at the C-terminal of a longer peptide are preferentially recognized [47]. Together our results suggest that the use of cohort-specific 10mer peptides is efficient at identifying epitopes, though the approach could be improved by the addition of consensus peptides to guarantee positional coverage across a protein.

As mosaic and multivalent vaccines continue to be evaluated in clinical trials [21,22,25], it will be increasingly important to measure the number of responses elicited and the extent to which the responses can cross-recognize circulating variants; one can improve the efficiency of this task by reducing the number of peptides needed to identify an individual’s epitopes, the bottleneck of many approaches is ultimately the number of T cells that are required [26]. We harnessed the high throughput screening efficiency of the ELISpot assay and maximized the use of precious clinical samples by employing an in-vitro polyclonal CD8 T cell expansion procedure from PBMC samples using a CD3/CD4 bi-specific monoclonal antibody and IL-2 [34,36,52,53]. The ELISpot assay was an optimized two-stage approach: (1) screen organized peptide pools using day-7 expanded CD8 T cells and (2) test deconvoluted individual peptides using day 10 expanded CD8 T cells all from a single vial of cryopreserved PBMC. This allowed the extensive analysis and characterization of CD8 T cell responses to 794 PTE HIV-1 Gag peptides with one sample of 10^7^ cryopreserved PBMCs enabling a direct comparison of approaches using consensus, global and cohort PTE peptides. Epitope mapping experiments are an invaluable tool for providing mechanistic insights into vaccine efficacy and viral control. By reducing the PBMC sample constraint of epitope mapping experiments and improving the efficiency of the peptide library through cohort-specific design it may be feasible to expand the coverage of epitope mapping experiments to larger regions of the HIV-1 genome including envelope, which is now commonly included in vaccine candidates.

## Figures and Tables

**Table 1:** Participants characteristics.

**Gender**	**Female**	18 *
	Male	12 *
**Age (years)**	Mean (SD)	34 ± 9
**Day post EDI**	Mean ± SD	348 ± 17
**CD4 Count (cells/uL)**	Mean ± SD	566 ± 198
**Plasma Viral load (copies/mL)**	Mean ± SD	8301 ± 6046
**Mode of infection**	Heterosexual discordant couples	
**HIV-1 subtype**	Subtype C	

*: Denotes number of participants of each gender.

## Data Availability

The data presented in this study are available on request from the corresponding author.

## Supplementary Materials

The following supporting information can be downloaded at: https://www.mdpi.com/article/10.3390/vaccines11020472/s1, Supplementary Figure S1: Epitope identification strategy; Supplementary Figure S2: Overall density of the lengths of the determined epitopes; Supplementary Figure S3: Analysis of quality of response induced by epitopes that required a non-consensus peptide and those that were detected by the consensus peptide; and Supplementary Figure S4: Distribution of the different length epitopes to the different epitope groupings.

## References

[1] Martins M.A., Shin Y.C., Gonzalez-Nieto L., Domingues A., Gutman M.J., Maxwell H.S., Castro I., Magnani D.M., Ricciardi M., Pedreño-Lopez N. (2017). Vaccine-induced immune responses against both Gag and Env improve control of simian immunodeficiency virus replication in rectally challenged rhesus macaques. PLoS Pathog..

[2] Andersson A.-M.C., Holst P.J. (2016). Increased T cell breadth and antibody response elicited in prime-boost regimen by viral vector encoded homologous SIV Gag/Env in outbred CD1 mice. J. Transl. Med..

[3] Schmitz J.E., Kuroda M.J., Santra S., Sasseville V.G., Simon M.A., Lifton M.A., Racz P., Tenner-Racz K., Dalesandro M., Scallon B.J. (1979). Control of Viremia in Simian Immunodeficiency Virus Infection by CD8 ^+^ Lymphocytes. Science.

[4] Walker-Sperling V.E., Pohlmeyer C.W., Veenhuis R.T., May M., Luna K.A., Kirkpatrick A.R., Laeyendecker O., Cox A.L., Carrington M., Bailey J.R. (2017). Factors Associated with the Control of Viral Replication and Virologic Breakthrough in a Recently Infected HIV-1 Controller. eBioMedicine.

[5] Rosenberg E.S., Altfeld M., Poon S.H., Phillips M.N., Wilkes B.M., Eldridge R.L., Robbins G.K., D’Aquila R.T., Goulder P.J.R., Walker B.D. (2000). Immune control of HIV-1 after early treatment of acute infection. Nature.

[6] Koofhethile C.K., Ndhlovu Z.M., Thobakgale-Tshabalala C., Prado J.G., Ismail N., Mncube Z., Mkhize L., van der Stok M., Yende N., Walker B.D. (2016). CD8 ^+^ T Cell Breadth and Ex Vivo Virus Inhibition Capacity Distinguish between Viremic Controllers with and without Protective HLA Class I Alleles. J. Virol..

[7] Betts M.R., Nason M.C., West S.M., De Rosa S.C., Migueles S.A., Abraham J., Lederman M.M., Benito J.M., Goepfert P.A., Connors M. (2006). HIV nonprogressors preferentially maintain highly functional HIV-specific CD8+ T cells. Blood.

[8] Borrow P., Lewicki H., Hahn B.H., Shaw G.M., Oldstone M.B. (1994). Virus-specific CD8+ cytotoxic T-lymphocyte activity associated with control of viremia in primary human immunodeficiency virus type 1 infection. J. Virol..

[9] Friedrich T.C., Valentine L.E., Yant L.J., Rakasz E.G., Piaskowski S.M., Furlott J.R., Weisgrau K.L., Burwitz B., May G.E., León E.J. (2007). Subdominant CD8 ^+^ T-Cell Responses Are Involved in Durable Control of AIDS Virus Replication. J. Virol..

[10] Migueles S.A., Laborico A.C., Shupert W.L., Sabbaghian M.S., Rabin R., Hallahan C.W., Van Baarle D., Kostense S., Miedema F., McLaughlin M. (2002). HIV-specific CD8+ T cell proliferation is coupled to perforin expression and is maintained in nonprogressors. Nat. Immunol..

[11] Carrington M., Bontrop R.E. (2002). Effects of MHC Class I on HIV/SIV Disease in Primates. AIDS.

[12] Dong T., Zhang Y., Xu K.Y., Yan H., James I., Peng Y., Blais M.-E., Gaudieri S., Chen X., Lun W. (2011). Extensive HLA-driven viral diversity following a narrow-source HIV-1 outbreak in rural China. Blood.

[13] Wood N., Bhattacharya T., Keele B.F., Giorgi E., Liu M., Gaschen B., Daniels M., Ferrari G., Haynes B.F., McMichael A. (2009). HIV Evolution in Early Infection: Selection Pressures, Patterns of Insertion and Deletion, and the Impact of APOBEC. PLoS Pathog..

[14] O’Connor D., Friedrich T., Hughes A., Allen T.M., Watkins D. (2001). Understanding cytotoxic T-lymphocyte escape during simian immunodeficiency virus infection. Immunol. Rev..

[15] Kwong P.D., Mascola J.R., Nabel G.J. (2012). The changing face of HIV vaccine research. J. Int. AIDS Soc..

[16] Nabel G.J., Kwong P.D., Mascola J.R. (2011). Progress in the rational design of an AIDS vaccine. Philos. Trans. R. Soc. B Biol. Sci..

[17] Rolland M., Nickle D.C., Mullins J.I. (2007). HIV-1 Group M Conserved Elements Vaccine. PLoS Pathog..

[18] Gaschen B., Taylor J., Yusim K., Foley B., Gao F., Lang D., Novitsky V., Haynes B., Hahn B.H., Bhattacharya T. (2002). Diversity Considerations in HIV-1 Vaccine Selection. Science.

[19] Novitsky V., Smith U.R., Gilbert P., McLane M.F., Chigwedere P., Williamson C., Ndung’U T., Klein I., Chang S.-Y., Peter T. (2002). Human Immunodeficiency Virus Type 1 Subtype C Molecular Phylogeny: Consensus Sequence for an AIDS Vaccine Design?. J. Virol..

[20] Yusim K., Kesmir C., Gaschen B., Addo M.M., Altfeld M., Brunak S., Chigaev A., Detours V., Korber B. (2002). Clustering Patterns of Cytotoxic T-Lymphocyte Epitopes in Human Immunodeficiency Virus Type 1 (HIV-1) Proteins Reveal Imprints of Immune Evasion on HIV-1 Global Variation. J. Virol..

[21] Santra S., Liao H.X., Zhang R., Muldoon M., Watson S., Fischer W., Theiler J., Szinger J., Balachandran H., Buzby A. (2010). Mosaic vaccines elicit CD8+ T lymphocyte responses that confer enhanced immune coverage of diverse HIV strains in monkeys. Nat. Med..

[22] Bakari M., Aboud S., Nilsson C., Francis J., Buma D., Moshiro C., Aris E.A., Lyamuya E.F., Janabi M., Godoy-Ramirez K. (2011). Broad and potent immune responses to a low dose intradermal HIV-1 DNA boosted with HIV-1 recombinant MVA among healthy adults in Tanzania. Vaccine.

[23] Frahm N., Kiepiela P., Adams S., Linde C.H., Hewitt H.S., Sango K., Feeney M.E., Addo M.M., Lichterfeld M., Lahaie M.P. (2006). Control of human immunodeficiency virus replication by cytotoxic T lymphocytes targeting subdominant epitopes. Nat. Immunol..

[24] Geldmacher C., Currier J.R., Herrmann E., Haule A., Kuta E., McCutchan F., Njovu L., Geis S., Hoffmann O., Maboko L. (2007). CD8 T-Cell Recognition of Multiple Epitopes within Specific Gag Regions Is Associated with Maintenance of a Low Steady-State Viremia in Human Immunodeficiency Virus Type 1-Seropositive Patients. J. Virol..

[25] Altfeld M., Addo M.M., Shankarappa R., Lee P.K., Allen T.M., Yu X.G., Rathod A., Harlow J., O’Sullivan K., Johnston M.N. (2003). Enhanced Detection of Human Immunodeficiency Virus Type 1-Specific T-Cell Responses to Highly Variable Regions by Using Peptides Based on Autologous Virus Sequences. J. Virol..

[26] Li F., Malhotra U., Gilbert P.B., Hawkins N.R., Duerr A.C., McElrath J.M., Corey L., Self S.G. (2006). Peptide selection for human immunodeficiency virus type 1 CTL-based vaccine evaluation. Vaccine.

[27] Zhao L., Zhang M., Cong H. (2013). Advances in the study of HLA-restricted epitope vaccines. Hum. Vaccines Immunother..

[28] Malhotra U., Li F., Nolin J., Allison M., Zhao H., Mullins J.I., Self S., McElrath M.J. (2007). Enhanced Detection of Human Immunodeficiency Virus Type 1 (HIV-1) Nef-Specific T Cells Recognizing Multiple Variants in Early HIV-1 Infection. J. Virol..

[29] Nielsen M., Lundegaard C., Blicher T., Lamberth K., Harndahl M., Justesen S., Røder G., Peters B., Sette A., Lund O. (2007). NetMHCpan, a Method for Quantitative Predictions of Peptide Binding to Any HLA-A and -B Locus Protein of Known Sequence. PLoS ONE.

[30] Landais E., Huang X., Havenar-Daughton C., Murrell B., Price M.A., Wickramasinghe L., Ramos A., Bian C.B., Simek M., Allen S. (2016). Broadly Neutralizing Antibody Responses in a Large Longitudinal Sub-Saharan HIV Primary Infection Cohort. PLoS Pathog..

[31] Amornkul P.N., Karita E., Kamali A., Rida W.N., Sanders E.J., Lakhi S., Price M., Kilembe W., Cormier E., Anzala O. (2013). Disease progression by infecting HIV-1 subtype in a seroconverter cohort in sub-Saharan Africa. AIDS.

[32] Kamali A., Price M.A., Lakhi S., Karita E., Inambao M., Sanders E.J., Anzala O., Latka M.H., Bekker L.-G., Kaleebu P. (2015). Creating an African HIV Clinical Research and Prevention Trials Network: HIV Prevalence, Incidence and Transmission. PLoS ONE.

[33] Tang J., Tang S., Lobashevsky E., Myracle A.D., Fideli U., Aldrovandi G., Allen S., Musonda R., Kaslow R.A., Zambia-UAB HIV Research Project, 2002 (2002). Favorable and unfavorable HLA class I alleles and haplotypes in Zambians predominantly infected with clade C human immunodeficiency virus type 1. J. Virol..

[34] Michelo C.M., Dalel J.A., Hayes P., Fernandez N., Fiore-Gartland A., Kilembe W., Tang J., Streatfield C., Gilmour J., Hunter E. (2020). Comprehensive epitope mapping using polyclonally expanded human CD8 T cells and a two-step ELISpot assay for testing large peptide libraries. J. Immunol. Methods.

[35] Hayes P., Fernandez N., Ochsenbauer C., Dalel J., Hare J., King D., Black L., Streatfield C., Kakarla V., Macharia G. (2021). Breadth of CD8 T-cell mediated inhibition of replication of diverse HIV-1 transmitted-founder isolates correlates with the breadth of recognition within a comprehensive HIV-1 Gag, Nef, Env and Pol potential T-cell epitope (PTE) peptide set. PLoS ONE.

[36] Wong J.T., Colvin R.B. (1991). Selective reduction and proliferation of the CD4+ and CD8+ T cell subsets with bispecific monoclonal antibodies: Evidence for inter-T cell-mediated cytolysis. Clin. Immunol. Immunopathol..

[37] Spentzou A., Bergin P., Gill D., Cheeseman H., Ashraf A., Kaltsidis H., Cashin-Cox M., Anjarwalla I., Steel A., Higgs C. (2010). Viral Inhibition Assay: A CD8 T Cell Neutralization Assay for Use in Clinical Trials of HIV-1 Vaccine Candidates. J. Infect. Dis..

[38] Carlson J.M., Schaefer M., Monaco D.C., Batorsky R., Claiborne D.T., Prince J., Deymier M.J., Ende Z.S., Klatt N.R., DeZiel C.E. (2014). Selection bias at the heterosexual HIV-1 transmission bottleneck. Science.

[39] Llano A., Williams A., Olvera A., Silva-Arrieta S., Brander C. (2013). Best-Characterized HIV-1 CTL Epitopes: The 2013 Update. HIV Mol. Immunol..

[40] Fiore-Gartland A., Manso B.A., Friedrich D.P., Gabriel E.E., Finak G., Moodie Z., Hertz T., De Rosa S.C., Frahm N., Gilbert P.B. (2016). Pooled-Peptide Epitope Mapping Strategies Are Efficient and Highly Sensitive: An Evaluation of Methods for Identifying Human T Cell Epitope Specificities in Large-Scale HIV Vaccine Efficacy Trials. PLoS ONE.

[41] Gill D.K., Huang Y., Levine G.L., Sambor A., Carter D.K., Sato A., Kopycinski J., Hayes P., Hahn B., Birungi J. (2010). Equivalence of ELISpot Assays Demonstrated between Major HIV Network Laboratories. PLoS ONE.

[42] Boaz M.J., Hayes P., Tarragona T., Seamons L., Cooper A., Birungi J., Kitandwe P., Semaganda A., Kaleebu P., Stevens G. (2009). Concordant Proficiency in Measurement of T-Cell Immunity in Human Immunodeficiency Virus Vaccine Clinical Trials by Peripheral Blood Mononuclear Cell and Enzyme-Linked Immunospot Assays in Laboratories from Three Continents. Clin. Vaccine Immunol..

[43] Waterhouse A.M., Procter J.B., Martin D.M.A., Clamp M., Barton G.J. (2009). Jalview Version 2—A multiple sequence alignment editor and analysis workbench. Bioinformatics.

[44] Kumar S., Stecher G., Tamura K. (2016). MEGA7: Molecular Evolutionary Genetics Analysis Version 7.0 for Bigger Datasets. Mol. Biol. Evol..

[45] Hertz T., Ahmed H., Friedrich D.P., Casimiro D.R., Self S.G., Corey L., McElrath M.J., Buchbinder S., Horton H., Frahm N. (2013). HIV-1 Vaccine-Induced T-Cell Reponses Cluster in Epitope Hotspots that Differ from Those Induced in Natural Infection with HIV-1. PLoS Pathog..

[46] Currier J.R., Robb M.L., Michael N.L., Marovich M.A. (2011). Defining epitope coverage requirements for T cell-based HIV vaccines: Theoretical considerations and practical applications. J. Transl. Med..

[47] Draenert R., Brander C., Xu G.Y., Altfeld M., Verrill C.L., Feeney M.E., Walker B.D., Goulder P.J. (2004). Impact of intrapeptide epitope location on CD8 T cell recognition: Implications for design of overlapping peptide panels. AIDS.

[48] Hemelaar J. (2013). Implications of HIV diversity for the HIV-1 pandemic. J. Infect..

[49] Paraskevis D., Hatzakis A. (2019). Global molecular epidemiology of HIV-1: The chameleon challenge. Lancet Infect. Dis..

[50] Stephenson K., Barouch D.H. (2013). A global approach to HIV-1 vaccine development. Immunol. Rev..

[51] Hulot S.L., Korber B., Giorgi E.E., Vandergrift N., Saunders K.O., Balachandran H., Mach L.V., Lifton M.A., Pantaleo G., Tartaglia J. (2015). Comparison of Immunogenicity in Rhesus Macaques of Transmitted-Founder, HIV-1 Group M Consensus, and Trivalent Mosaic Envelope Vaccines Formulated as a DNA Prime, NYVAC, and Envelope Protein Boost. J. Virol..

[52] Hayes P.J., Cox J.H., Coleman A.R., Fernandez N., Bergin P.J., Kopycinski J.T., Nitayaphan S., Pitisuttihum P., de Souza M., Duerr A. (2016). Adenovirus-based HIV-1 vaccine candidates tested in efficacy trials elicit CD8+ T cells with limited breadth of HIV-1 inhibition. AIDS.

[53] Pira G.L., Ivaldi F., Moretti P., Manca F. (2010). High Throughput T Epitope Mapping and Vaccine Development. J. Biomed. Biotechnol..

