# Supplementary material for: Cohort-Specific Peptide Reagents Broaden Depth and Breadth Estimates of the CD8 T Cell Response to HIV-1 Gag Potential T Cell Epitopes
Source: Vaccines (Basel). 2023 Feb 17;11(2):472. doi: 10.3390/vaccines11020472 (PMC9961105; doi:10.3390/vaccines11020472)
Supplement: Supplementary file 1 [file vaccines-11-00472-s001.zip › vaccines-2204111-supplementary.pdf]

Supplementary Figures

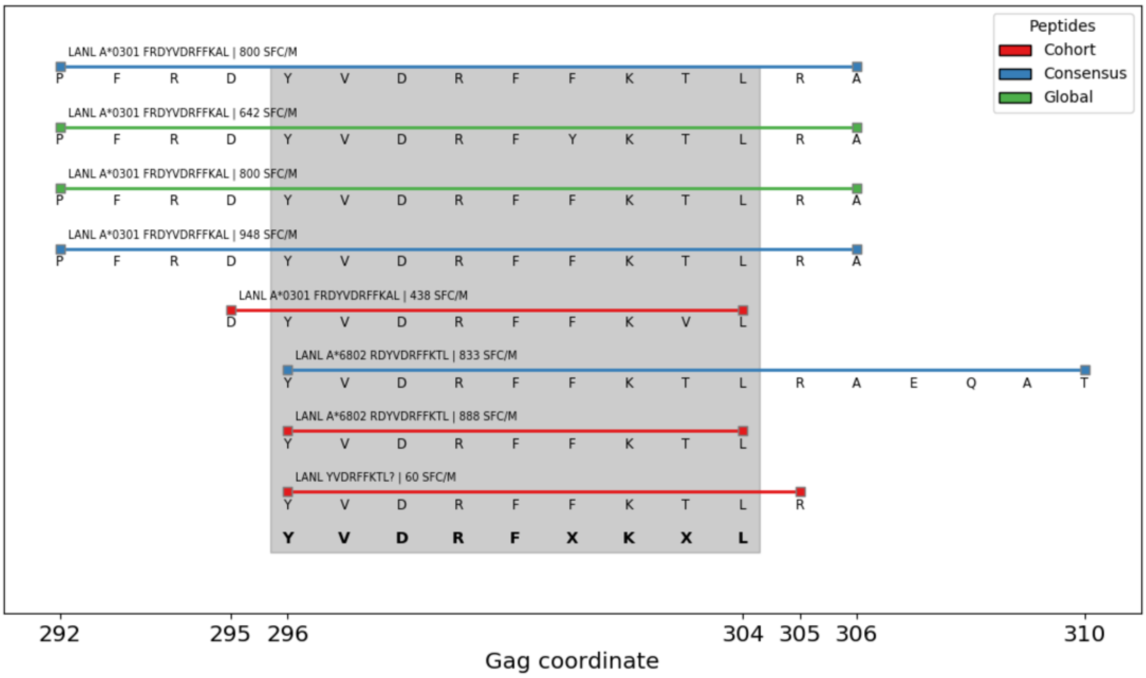

Supplementary Figure S1 Epitope identification. Multiple but distinct overlapping peptides from a single participant were considered to reflect a response to one underlying epitope. Thus, to identify the minimal set of underlying epitopes that could explain each participant's peptide responses, we applied an overlap criterion that if two or more peptides share a region of ≥8 positions then they can be explained by a single epitope. This criterion was applied iteratively to all possible combinations of a patient's peptide responses, to identify the minimal number of epitopes that could explain all the peptide responses.

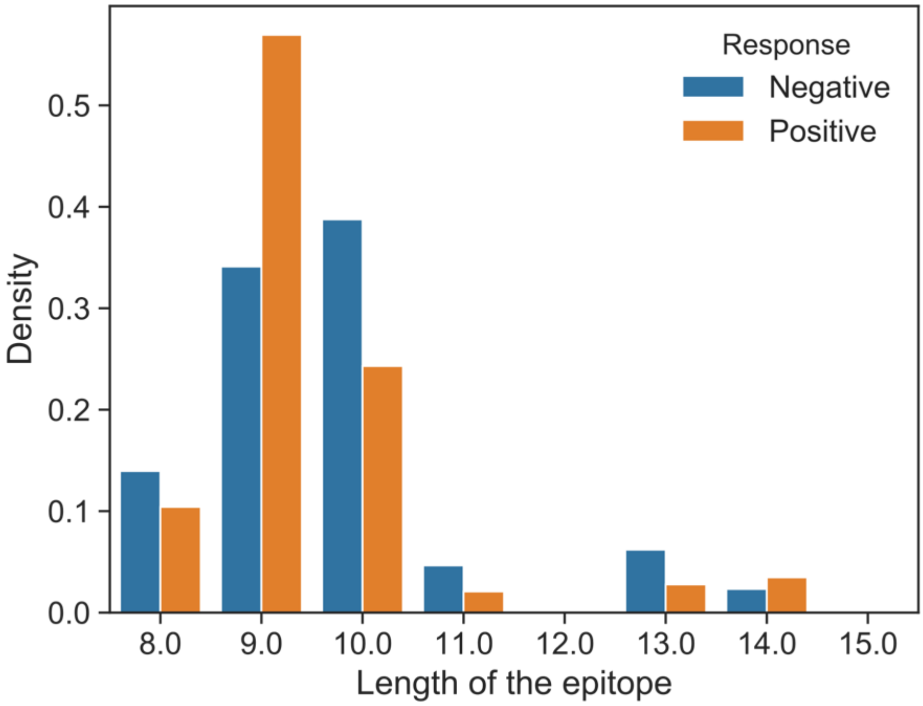

Supplementary Figure S2 Overall density of the lengths of the determined epitopes. Comparison made between the densities in the positive and negative peptides

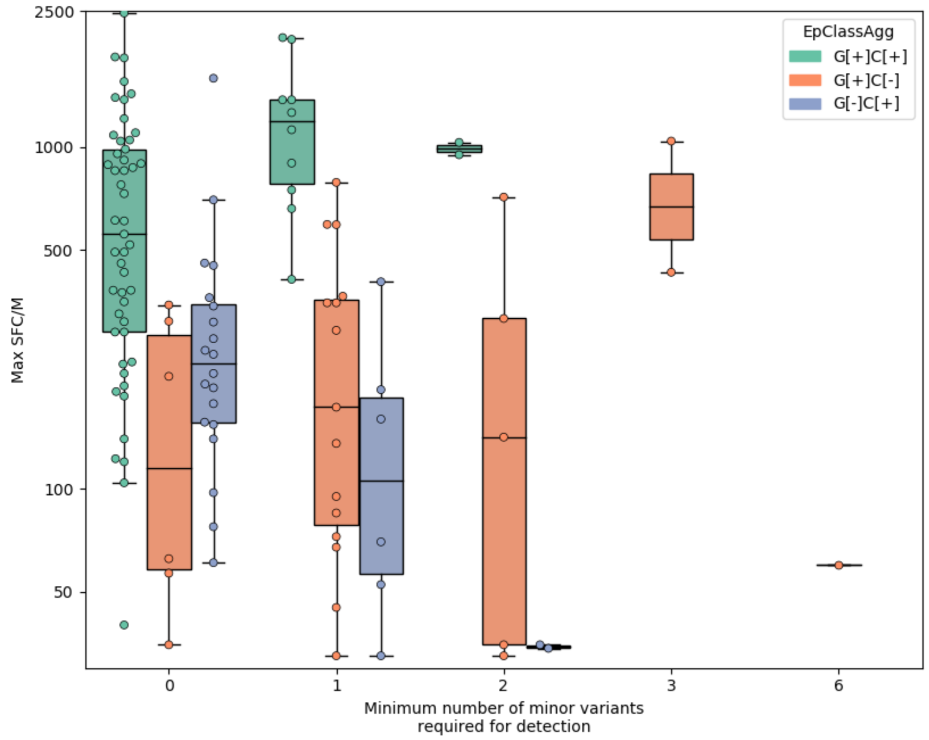

Supplementary Figure S3. Analysis of quality of response induced by epitopes that required a non-consensus peptide and those that were detected by the consensus peptide

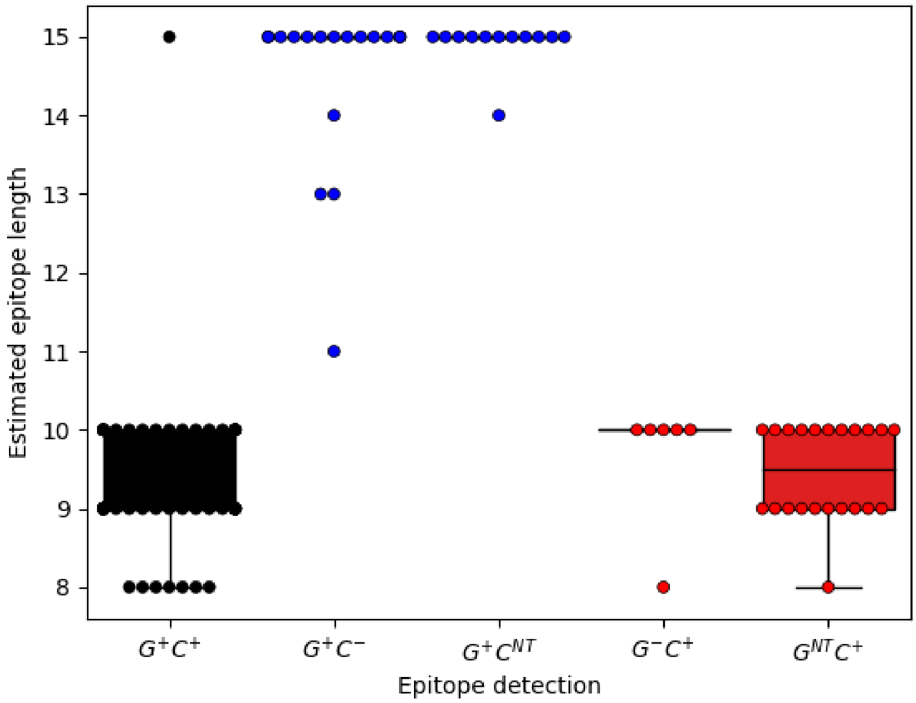

Supplementary Figure S4 Distribution of the different length epitopes to the different epitope groupings
